# The Genetics of Tuberous Sclerosis Complex and Related mTORopathies: Current Understanding and Future Directions

**DOI:** 10.3390/genes15030332

**Published:** 2024-03-04

**Authors:** Alice Man, Matteo Di Scipio, Shan Grewal, Yujin Suk, Elisabetta Trinari, Resham Ejaz, Robyn Whitney

**Affiliations:** 1 Michael G. DeGroote School of Medicine, McMaster University, Hamilton, ON L8S 4L8, Canada; 2 Division of Developmental Pediatrics, Department of Pediatrics, McMaster Children’s Hospital, Hamilton, ON L8N 3Z5, Canada; 3 Division of Genetics, Department of Pediatrics, McMaster University, Hamilton, ON L8S 4L8, Canada; 4 Division of Neurology, Department of Pediatrics, McMaster University, Hamilton, ON L8S 4L8, Canada

**Keywords:** genetic epilepsies, mTORopathies, tuberous sclerosis complex, precision medicine, focal cortical dysplasia, genetic testing, malformations of cortical development, mosaicism

## Abstract

The mechanistic target of rapamycin (mTOR) pathway serves as a master regulator of cell growth, proliferation, and survival. Upregulation of the mTOR pathway has been shown to cause malformations of cortical development, medically refractory epilepsies, and neurodevelopmental disorders, collectively described as mTORopathies. Tuberous sclerosis complex (TSC) serves as the prototypical mTORopathy. Characterized by the development of benign tumors in multiple organs, pathogenic variants in *TSC1* or *TSC2* disrupt the TSC protein complex, a negative regulator of the mTOR pathway. Variants in critical domains of the TSC complex, especially in the catalytic TSC2 subunit, correlate with increased disease severity. Variants in less crucial exons and non-coding regions, as well as those undetectable with conventional testing, may lead to milder phenotypes. Despite the assumption of complete penetrance, expressivity varies within families, and certain variants delay disease onset with milder neurological effects. Understanding these genotype–phenotype correlations is crucial for effective clinical management. Notably, 15% of patients have no mutation identified by conventional genetic testing, with the majority of cases postulated to be caused by somatic *TSC1/TSC2* variants which present complex diagnostic challenges. Advancements in genetic testing, prenatal screening, and precision medicine hold promise for changing the diagnostic and treatment paradigm for TSC and related mTORopathies. Herein, we explore the genetic and molecular mechanisms of TSC and other mTORopathies, emphasizing contemporary genetic methods in understanding and diagnosing the condition.

## 1. Introduction

Tuberous sclerosis complex (TSC) is a multisystemic genetic disorder characterized by the development of benign tumors in various organs, stemming from overactivation of the mechanistic target of rapamycin (mTOR) pathway. TSC is inherited in an autosomal dominant pattern in approximately one third of cases. De novo and/or mosaic pathogenic variants in *TSC1* and *TSC2* account for the other two thirds of TSC genetic etiology. The incidence of TSC ranges between 1:5800 to 1:13,520 live births [1,2].

The condition is marked by the development of non-cancerous growths or hamartomas in multiple organs, with particular clinical attention given to neurological presentations such as epilepsy and neuropsychiatric disorders that vary from infancy to adulthood [3]. TSC-related mortality is predominantly linked to epilepsy-related complications, particularly status epilepticus and sudden unexpected death in epilepsy, as well as TSC-associated renal complications [4,5]. Clinical presentation is complex where inter-individual variability in phenotypic severity contributes to gaps in understanding the natural progression of TSC. Even within families, there can exist a phenotypic range from normal cognitive development and well-managed epilepsy to instances of severe intellectual disability, medically refractory epilepsy, and autism spectrum disorder (ASD) [6]. A major key to understanding the variable clinical spectrum of TSC is through continuing to elucidate the mechanistic roles of *TSC1*/*TSC2* in the mTOR pathway and the genotype–phenotype correlations of associated genetic variants.

Pathogenic variants in *TSC1* or *TSC2* inactivate key inhibitors of the mTOR pathway, which mediate cell growth and metabolism. Consequently, upregulated mTOR leads to cellular overgrowth and tumor formation, and the brain is often the most severely affected organ [2]. More generally, pathogenic variants in other mTOR regulators can lead to similar neurodevelopmental disorders, as in TSC, collectively known as mTORopathies. To date, over 16 causative genes are known, including *TSC1* and *TSC2* [7,8]. This review discusses the genetic and molecular mechanisms of TSC and mTORopathies, with a particular focus on contemporary genetic methods to identify and manage the neurological and neurodevelopmental aspects associated with the condition.

## 2. Overview of the Genetics of mTORopathies

mTORopathies encompass a spectrum of neurodevelopmental disorders including TSC, focal cortical dysplasia type II (FCD II), hemimegalencephaly (HME), and Pretzel syndrome (polyhydramnios, megalencephaly, and symptomatic epilepsy; PMSE syndrome) [9]. Next-generation sequencing platforms have led to the identification of a common genetic etiology to a subset of malformations of cortical development—upregulation of the mTOR signaling pathway [10]. Notably, recent findings have distinguished FCD I from FCD II and other mTORopathies in which FCD I was found to be associated with glycosylation defects of *SLC35A2* rather than the mTOR pathway [11]. mTORopathies share common neuropathological findings including abnormal cellular morphology and cytomegaly, disorganized cortical lamination, and neuronal hyperexcitability resulting from constitutive mammalian target of rapamycin complex 1 (mTORC1) signaling [8].

Although the exact pathobiology of mTORopathies has yet to be elucidated, mTOR is known to play a role in the brain in controlling cortical development and neural-specific functions including synaptic plasticity, learning, and memory. Thus, mTOR dysfunction is implicated in several neurodevelopmental, neurodegenerative, and psychiatric disorders [9]. With regard to epileptogenesis, mTORC activation is thought to disrupt the formation of proper neural circuits by altering normal neural networks, resulting in excitatory/inhibitory imbalance and net synaptic hyperexcitability [8,12]. Notably, in a rodent biallelic model of *TSC1* deletion, mTORC activation alone without significant cortical malformations was sufficient to generate seizures [13].

The mechanistic target of rapamycin complex (mTORC) serves as a master regulator of cell growth, proliferation, and survival, ultimately governing cell fate decisions and regulating biomass accumulation and cellular metabolism processes [14]. The mTOR protein is part of both mTORC1 and mTORC2 complexes, serving as the central node that integrates information regarding availability of energy and nutrients to coordinate the synthesis (anabolism) or breakdown (autophagy) of the essential cellular building blocks [14]. mTORC1 is nucleated by three core components, mTOR, mLST8, and RAPTOR, while mTORC2 is composed of mTOR, mLST8, and RICTOR. During times of abundance, mTORC1 phosphorylates downstream substrates that increase production of lipids, proteins, nucleotides, and ATP while inhibiting catabolism and autophagy pathways [15].

The canonical mTORC1 signaling pathway involves upstream signaling from extracellular growth factors binding to their respective receptor tyrosine kinases (RTKs) and initiating downstream signaling pathways (i.e., PI3K-AKT and ERKs). Upon activation, AKT and ERKs phosphorylate TSC2 to release it from the TSC1/TSC2 complex, preventing TSC2 from inhibiting RHEB, a direct activator of mTORC1. Following mTORC1 activation, pivotal signaling effectors such as S6K and 4EBP1 are phosphorylated to drive cellular response such as protein synthesis, energy metabolism, and inhibition of autophagy [9]. mTORC1 is also negatively regulated by an upstream protein complex GATOR1, composed of three subunits DEPDC5, NPRL2, and NPRL3, that represses the mTORC1 complex in response to amino acid depletion. Similarly, the KICSTOR complex, composed of KPTN, ITFG2, C12orf66, and SZT2, recruits the GATOR1 complex to the lysosomal surface to inhibit mTORC1 [16]. Pathogenic variants in any of the GATOR1 or KICSTOR subunits can also contribute to mTORopathy development and manifestation of neurological symptoms, such as epilepsy, malformations of cortical development (i.e., FCDs), and neurodevelopmental disorders [8].

mTORC signaling plays a large role in neural development and regulation; therefore, pathogenic variants along the mTOR pathway have profound effects on the central nervous system. mTORopathies generally stem from pathogenic variants in the activators of the mTOR pathway (*PI3KCA*, *AKT3*, *RHEB*, *MTOR*) or loss of function variants in inhibitors of mTOR (*TSC1*, *TSC2*, *DEPDC5*, *NPRL3*, *NPRL2*, *PTEN*, *STRADA*) that result in constitutive activation of the mTORC1 complex [8] (Table 1).

Both somatic and germline pathogenic variants have been implicated in the development of mTORopathies and can define the extent of clinical manifestations depending on the origin of the variant along the embryological timeline [17]. For instance, HME and FCD II are thought to exist on the same disorder continuum with their distinction defined by the relative size/volume of affected lesions. HME stems from an earlier onset of the postzygotic somatic variant (thereby affecting a greater number of subsequent clones) during neuroepithelium maturation, resulting in the involvement of an entire hemisphere or even including the ipsilateral cerebellar hemisphere and brainstem in “total HME” [12]. On the other hand, FCD II involves more focal lesions often involving a single gyrus or several adjacent gyri or part of one hemisphere, stemming from genetic variants occurring at later mitotic stages (fewer subsequent clones thereby spanning fewer developmental regions) [12].

Due to the diverse phenotypic heterogeneity between mTORopathy subtypes and even within specific disorders, magnetic resonance imaging (MRI) findings can vary widely [8]. HME and megalencephaly (ME) show distinct cortical abnormalities as they involve whole brain (ME) and hemispheric (HME) abnormalities, while FCD can be missed on initial MRI and often requires high-resolution scanning [3]. The pathobiology behind the involvement of multiple organs (i.e., TSC) or brain-specific disorders (i.e., FCD II, HME) is not as clearly defined but may relate to organ-specific gene expression, timing of the mutagenesis, and the nature of each unique molecular driver [8]. While some mTORopathies can affect multiple organs in patients, neuropsychiatric disorders, including epilepsy, intellectual disability, and autism spectrum disorder, resulting from the cortical malformations tend to have the most impact on quality of life.

**Table 1:** Genes associated with mTORopathies, their role in the mTOR pathway, variant type, and clinical manifestations.

Gene	Major Affected Sensing Arm(s) of mTOR Pathway	Previously Reported Genetic Variant Type(s)	CNS Pathological Manifestations	Reported Clinical Manifestations and Syndromes	Systemic Features Present *
**Variants of mTOR Activators**					
*MTOR* [18,19]	Amino acid, growth factor, cytokine signaling, cellular energy, and oxygen stress [20]	Somatic and germline	FCD Hemimegalencephaly Megalencephaly	Epilepsy Smith–Kingsmore syndrome Intellectual disability	(+)
*AKT1* [21,22]	AKT family: growth factor [23]	Somatic	Hemimegalencephaly	Epilepsy Motor weakness Intellectual disability Somatic Proteus syndrome	(+)
*AKT3* [24,25]	AKT family: growth factor [23]	Somatic and germline	Hemimegalencephaly Megalencephaly Polymicrogyria	Epilepsy MPPH syndrome Intellectual disability	(-)
*PIK3CA* [21,26]	PI3K family: growth factor [20]	Somatic and germline	Hemimegalencephaly Megalencephaly Dysplastic megalencephaly FCD	Epilepsy PIK3CA-related overgrowth spectrum Intellectual disability	(+)
*PIK3R2* [25,27]	PI3K family: growth factor [20]	Somatic and germline	Megalencephaly	Epilepsy MPPH Intellectual disability	(-)
*RHEB* [28]	Amino acid, growth factor [29]	Somatic	FCD Hemimegalencephaly	Epilepsy	(-)
**Loss of Function Variants of mTOR Inhibitors**					
*TSC1/TSC2* [2]	TSC complex: amino acid, growth factor, cytokine signaling, cellular energy and oxygen, stress [20,30]	Somatic and germline	Cortical tubers SEN SEGA Hemimegalencephaly FCD	Epilepsy Tuberous sclerosis complex Intellectual disability	(+)
*TBC1D7* [31,32]	Forms part of TSC complex	Germline	Macrocephaly/megalencephaly	Intellectual disability	(+)
*DEPDC5* [33,34,35,36]	GATOR1 complex: amino acid [20,37]	Somatic and germline	Hemimegalencephaly Megalencephaly FCD	Epilepsy Intellectual disability Psychiatric comorbidities	(-)
*NPRL2* [38,39]	GATOR1 complex: amino acid [20,37]	Somatic and germline	FCD	Epilepsy Intellectual disability Psychiatric comorbidities	(-)
*NPRL3* [38,39,40]	GATOR1 complex: amino acid [20,37]	Somatic and germline	FCD	Epilepsy Intellectual disability Psychiatric comorbidities	(-)
*PTEN* [41,42,43,44]	Growth factor, cellular metabolism [45]	Somatic and germline	FCD Hemimegalencephaly Megalencephaly Polymicrogyria	Focal epilepsy ASD Neurodevelopmental disorders Cowden syndrome	(+)
*STRAD*α [46,47]	Cellular energy [20]	Germline	Megalencephaly	Focal epilepsy Pretzel syndrome Intellectual disability	(+)
*SZT2* [48,49]	KICSTOR complex: amino acid [50]	Germline	Megalencephaly	Developmental delay Epileptic encephalopathy	(-)
*KPTN* [51,52,53]	KICSTOR complex: amino acid [50]	Germline	Macrocephaly/Megalencephaly	Epilepsy Intellectual disability	(-)

* (+) denotes the report of at least one systemic feature (beyond CNS manifestations) in at least one individual; (-) denotes no non-CNS manifestations. (-) could include reports of facial dysmorphisms and minor skeletal dysplasias but were generally reported to be non-syndromic. Abbreviations: FCD: focal cortical dysplasia. SENs: subependymal nodules. SEGA: subependymal giant cell astrocytoma. MPPH: megalencephaly-polymicrogyria-polydactyly-hydrocephalus. ASD: autism spectrum disorder.

## 3. Overview of Genetics of TSC

TSC is the prototypical mTORopathy, caused by inactivating pathogenic variants in the *TSC1* and *TSC2* genes. *TSC1* occurs on the distal q-arm of chromosome 9 (9q34) and includes 23 exons (hg38: g.9:132891348-132946874; NM_000368.5). *TSC1* codes for the TSC1 protein (or hamartin), which is 1,164 amino acids long (130 kDa) [54]. *TSC1* comprises an N-terminal α-helical ‘core’ domain and a coiled coil located at the C-terminus, essential for its interaction with TSC2 [55]. Functional studies have demonstrated the importance of coding regions in the N-terminal domain, specifically exons 4–9, as well as exon 18, for TSC1 function and stability. Conversely, exons 10, 12, 14, and 16 have been shown to have the least effect on stability [56]. This aligns with up-to-date reports of pathogenic or likely pathogenic missense variants in *TSC1*, where TSC causative missense variants cluster on exons 2, 3, 7, and 8 encoding the N-terminal domain, as opposed to the latter exons [57]. However, it should be noted that missense variants account for only 6% of *TSC1* pathogenic variants. Small indels account for over 57% of all pathogenic variants [2], while nonsense variants, splice variants, large deletions, and rearrangements account for the remaining 37%. Overall, while most pathogenic variants are distinct, those in specific codons of exon 15 have been known to recur [2].

*TSC2* is located on the distal p-arm of chromosome 16 (16p13.3) and includes 42 exons (hg38: g.16:2047985-2089491; NM_000548.5). TSC2 (or tuberin) is 1807 amino acids long (200 kDa), comprising an N-terminal α-solenoid domain that binds to TSC1, as well as a coiled coil at the C-terminal domain with important functional motifs [55]. These motifs include the GAP domain, estrogen receptor- and calmodulin-binding domains, and multiple signal pathway kinase targets [2]. Pathogenic missense variants are more frequent in *TSC2* compared with *TSC1*, accounting for approximately 26% of all pathogenic variants. Approximately half of reported pathogenic missense variants encode the C-terminal domain [2,58,59]. Small indels account for 38% of pathogenic variants, while nonsense and splice variants, large deletions, and rearrangements account for the remaining 36% [2,58].

To date, 984 unique pathogenic or likely pathogenic variants in *TSC1* and 2832 in *TSC2* have been reported in the Leiden Open Variation Database version 3 [60]. These variants are estimated to be inherited in an autosomal dominant manner in one-third of TSC patients and arise de novo in the remaining two-thirds [58]. While pathogenic variants are found through conventional genetic testing in approximately 85% of individuals with TSC, no mutations are identified (NMI) for 10-15% of TSC patients who are diagnosed by clinical criteria [6,61,62,63,64]. Hypothesized reasons for NMI are (1) mosaicism in *TSC1* or *TSC2*, (2) pathogenic variants in deep intronic regions, or (3) variants in an unknown gene (i.e., *TSC3*) [65]. 

Genetic mosaicism is caused by postzygotic somatic pathogenic variants that arise after fertilization. These variants impart genomic diversity across tissues, with the coexistence of genetically distinct cell populations within the organism. If a subset of somatic tissues is affected, the variant is termed a “somatic mosaic variant” or “somatic variant”; if a subset of germline cells is affected, the variant is termed a “germline or gonadal mosaic variant”. A “gonosomal mosaic variant” affects both somatic and germline cell types [66,67]. The number of tissues affected depends on the timing of the initial variant: pathogenic variants occurring early in embryonic development are likely to affect more tissues, while those occurring later are likely to be localized to a single organ. For example, mosaic variants found in both brain and blood may have arisen prior to ectoderm and mesoderm formation [68]. In the context of TSC, mosaicism presents a challenge for genetic testing as the genetic variant responsible for the condition may not be present in all clinically accessible tissue. Relying solely on a blood or skin sample may result in false negatives if the pathogenic variant is confined to specific tissues (e.g., skin, brain) and not represented elsewhere. Even if the mosaic variant is present in blood, the variant may still not be detected, due to technical limitations of the genetic tests used [69]. This is particularly true if the mosaic cell population only comprises a small proportion of the sample, namely, if the variant allele frequency (VAF) is low. A high read depth, or more sequencing reads per base, is required to detect variants with low VAF. Sequencing pipelines to detect variants with VAF of <10 to 20% are not typically available in the clinical setting [70].

Pathogenic variants in deep intronic regions are also suspected to contribute to NMI status. Clinical genetic testing typically includes only cytogenetic tests, such as chromosomal microarrays, gene panels catered to specific phenotypes, and next-generation sequencing (NGS) approaches, such as exome sequencing (ES) and genome sequencing (GS). At the present, ES is more commonly used in the clinic compared with GS, leading to variants in non-coding sequences being missed. While GS has the ability to detect intronic variants, the increase in diagnostic yield has remained modest for many patient cohorts [71]. This can be attributed to challenges in interpreting intronic variants and in identifying candidate genes that they are likely to affect. While splicing prediction tools are available for use, they are best for identifying the impact of canonical splice site variants [72]. Newer tools, such as SpliceAI, are proving to be more sensitive for detecting deep intronic variants [73]. Many recent studies have also shown the utility in re-phenotyping and re-genotyping cases with NMI, where deep intronic variants have improved the diagnostic yield, including a recent study on *TSC1/TSC2* [74,75,76]. Most deep intronic variants will require functional studies for their pathogenicity to be evaluated as per guidelines from the American College of Medical Genetics and Genomics (ACMG) and Association for Molecular Pathology (AMP) [71,77,78].

Multiple studies have been reported in support of the two aforementioned hypotheses. Mosaic and intronic *TSC1/TSC2* variants have been identified in up to 58% and 40% of NMI individuals, respectively [65,79]. While most studies do not support the presence of a third TSC gene, a recent case study has proposed that variants in *RHEB* may explain some NMI cases. A study described a child meeting the clinical criteria for a TSC diagnosis without pathogenic variants in *TSC1*/*TSC2* but with a pathogenic somatic variant in *RHEB* at 13% VAF in a brain-derived sample [28]. He presented with focal seizures, tubers with radial migration lines, subependymal nodules (SEN), and a subependymal giant cell astrocytoma (SEGA), wherein three major criteria were met (only two are required) for the diagnosis of TSC. While many genes in the mTOR pathway, including *RHEB*, have been shown to cause other malformations of cortical development (MCDs) [11], they have not been associated with the TSC phenotype until this report. Although this patient may have an additional undetected germline variant, such as a deep intronic variant affecting *TSC1/TSC2*, this report suggests the importance of continuing to explore other mTOR pathway genes to understand their contribution to the TSC phenotype.

## 4. Summary of Genotype–Phenotype Correlations for TSC

Both TSC1 and TSC2 are essential for the optimal functioning of the TSC protein complex. From a structural perspective, genetic variants affecting important protein domains, such as those in the catalytic TSC2 subunit, are likely to correlate with greater disease severity. Conversely, variants in less critical exons and non-coding regions, as well as those unidentifiable with conventional genetic testing, are likely to present with a less severe phenotype [80]. While variants are thought to display complete penetrance, their expressivity can differ greatly between individuals, even within the same family [6]. There is also thought to be a subset of variants where the time of disease onset is delayed and neurological effects are milder [81,82,83]. Thus, a more nuanced understanding of genotype–phenotype correlations in TSC is valuable for clinical management.

Several important genotype–phenotype correlations have been observed for TSC. First and foremost, *TSC2* pathogenic variants have been shown to result in more severe phenotypic outcomes compared with those in *TSC1* [62,64,84]. This phenotypic correlation is consistent with TSC2 being the catalytically active subunit and TSC1 being the stabilizing subunit with proposed functions such as prevention of TSC2 ubiquitination [56,85,86]. In addition, simplex cases are more likely to involve *TSC2* pathogenic variants, while inherited pathogenic variants are more evenly split between *TSC1* and *TSC2* [64]. Furthermore, *TSC2* pathogenic variant carriers are at greater risk for renal malignancies, cardiac rhabdomyomas, intellectual disability, infantile epileptic spasms, and drug-resistant epilepsy [81,87,88,89]. In infancy, up to 24 months of age, *TSC2* pathogenic variant carriers are at higher risk for significant developmental delays [90]. *TSC2* pathogenic variants are also more frequently associated with positive TSC-associated neuropsychiatric disorders (TAND) such as attention deficit hyperactivity disorder (ADHD) [91] and ASD [92]. TSC-associated ASD has been shown to be associated with SEGA and cystic tuber formation [92,93]. The frequency, severity, and age of onset of epilepsy is also higher and younger in *TSC2* patients [94], and these findings have been replicated in mouse models [95]. Likewise, the surgical outcomes of epilepsy are less responsive in those with *TSC2* compared with *TSC1* variants [96]. While mitochondrial DNA (mtDNA) variants were initially hypothesized to be a potential cause of phenotypic variability in TSC, a recent study showed that the mitochondrial genome is highly stable across tissues and within TSC-associated tumors, without correlations to any TSC clinical features [97]. 

The types of genetic variants in *TSC1*/*TSC2* also correlate with clinical severity. There are many instances of milder phenotypes occurring in either gene [17]. A study showed that patients with premature protein truncations were more likely to manifest major symptoms of the diagnostic criteria (SEN, cortical tubers, and renal cysts) than patients without premature protein truncations [98,99]. As NGS approaches have become more widespread, a growing proportion of pathogenic variants have been reported as copy number variants (CNVs). In particular cohorts, this represents up to 10% of variants [100,101]. In neurological tissue, intronic pathogenic variants that cause aberrant exonization have been associated with milder phenotypes (sometimes with only one clinical feature present in a systemic disease) hypothesized to be explained by significantly abrogated, but not eliminated, canonical transcript expression [76,102,103]. In a recent cohort study reevaluating NMI TSC patients with a definite or possible clinical TSC diagnosis, 19/155 individuals presented with deep intronic variants; of these intronic carriers, several of them did not have a definite clinical diagnosis nor did they display as many systemic features as did the rest of the cohort [74].

## 5. Updates to Genotype–Phenotype Correlations in TSC: Mosaicism and the Two-Hit Hypothesis

TSC patients with NMI and mosaic variants have been found to have a milder clinical phenotype compared with those with constitutional germline variants [64,65,104]. In a study of 53 NMI TSC patients, those with previously missed germline variants were found to have an average of six major TSC symptoms, while those with mosaic variants and persistent NMI presented, on average, with only four and three major symptoms, respectively [65]. Similarly, a recent study of 95 NMI individuals showed that the total number of TSC clinical features was lower in those with mosaicism compared with those with previously missed germline variants, as well as those in an age- and sex-matched cohort from the TSC Natural History Database (NHD) [105]. No differences were found in the numbers of total or organ-specific clinical features in individuals with mosaic variants in *TSC1* versus *TSC2*. Interestingly, fibrous cephalic plaques and renal angiomyolipomas (AMLs) were both significantly more common in mosaic individuals compared with those with germline variants [105]. A significant positive correlation was also found between median VAF in blood and/or saliva and the number of TSC clinical features, for both *TSC1* and *TSC2*. Namely, those with higher VAF in clinically accessible samples were more likely to present with a greater number of TSC features [105]. However, the opposite was found in another study of 31 NMI TSC patients, where blood, saliva, and buccal VAF in the 16 mosaic patients were not associated with the number of major clinical features [106]. Yet, the study did find that VAFs from saliva and buccal samples correlated with tuber load.

Limited evidence exists as to whether there is a VAF threshold for clinical manifestations of TSC to become evident. Overall, for mTORopathies causing MCD, there does not seem to be a clear threshold, based on the current limits of detection. Variants affecting the mTOR pathway have been found with VAFs of as low as 1% in brain tissue resected from individuals with epilepsy and are thought to be a cause of disease [107,108]. However, it is unclear whether mosaicism in isolated brain cell populations or even lower VAFs in the brain cause MCD or associated symptomatology [66]. In clinically accessible samples, VAFs do not always correlate with those in affected tissue such as the brain, as organs affected depend on the embryonic lineage affected as well as the timing of the initial variant [65,106,109,110,111]. This can be exemplified by a case study in which two individuals with *TSC2* VAFs of ≤25% in blood showed severe, multiorgan TSC phenotypes [112]. Additionally, TSC patients with *TSC2* mosaic variants were found to have VAFs of as low as 0.21% in clinically accessible samples [65]. Interestingly, for *TSC1*, there is evidence suggesting that a VAF threshold exists in clinically accessible samples for the manifestation of TSC, potentially due to the milder effect of *TSC1* variants overall. Klonowska and colleagues proposed that *TSC1* individuals with VAF < 3% in blood or saliva could be less likely to meet clinical diagnosis criteria. This is due to their findings that (1) individuals with mosaic *TSC1* variants had higher VAFs (median of 7.74%) compared with those with mosaic *TSC2* (median of 1.93%) and (2) the proportion of mosaic *TSC1* to *TSC2* variants in their cohort was lower than that seen in the general TSC population [105]. 

Another complexity arising from the interpretation of mosaic pathogenic variants is their role in the diagnosis of TSC. Currently, a diagnosis of TSC can be established using clinical diagnostic criteria, namely, whether the individual presents with a minimum of two major features or one major feature with two minor features [2]. A molecular diagnosis can also be established if a heterozygous pathogenic variant is detected in *TSC1/TSC2*, regardless of clinical findings. Mosaic variants have been detected in individuals with clinical TSC diagnoses as aforementioned and have been assumed to be the cause of the disease. However, the line becomes blurred when mosaic pathogenic variants are detected in *TSC1/TSC2* and the individual does not meet clinical diagnostic criteria for TSC. Isolated mosaicism in *TSC1/TSC2* in the brain has been shown to result in FCD or HME without systemic manifestations of TSC, or neurologic manifestations typical of TSC such as SENs and SEGAs [11,28,107,113,114]. Similarly, patients with a progressive lung disease, lymphangioleiomyomatosis (LAM), can have sporadic LAM (S-LAM) with *TSC1/TSC2* variants, probably two somatic hits, isolated to the affected tissue [115,116,117]. The two-hit hypothesis, first described by Dr. Alfred Knudson in 1971, claims that both alleles of a tumor suppressor gene must be inactivated for the initiation and subsequent formation of tumors or lesions. In the case of an inherited germline variant present in one allele, the second copy must also then be somatically mutated (loss of heterozygosity) for the formation of the tumor or phenotypic expression of the lesion [118]. Cases with two somatic hits do not warrant a diagnosis of TSC, as surveillance for other progressive systemic manifestations that comes with the diagnosis is less necessary. 

However, care must be taken to ensure that the mosaic variant is truly isolated within the organ affected. A recent case study showed that three individuals thought to have S-LAM were diagnosed with mosaic TSC-associated LAM upon finding *TSC* variants in an angiofibroma, cutaneous hamartoma, and a digit affected by macrodactyly, respectively [117]. These individuals probably had systemic mosaicism in *TSC1/TSC2* and a second hit in LAM tissue. They were therefore subsequently diagnosed with TSC due to the importance of surveillance for progressive systemic TSC traits. Conversely, an individual with widespread clinical manifestations of TSC (renal AMLs, angiofibromas, fibrous cephalic plaque, enamel pits, retinal hamartomas, cortical tubers, and SEN) was found to have an isolated somatic *TSC2* pathogenic variant in renal AML tissue, with none in skin fibroblasts or saliva [116]. This could be explained by the two-hit hypothesis, where another systemic mosaic variant or germline variant was present but undetected. This demonstrates that even if a mosaic *TSC1/TSC2* variant is thought to be isolated within a specific tissue type, additional undetected variants could affect the individual’s phenotype. Systemic manifestations cannot be fully ruled out in individuals with an initial presentation of isolated *TSC1/TSC2* somatic pathogenic variants.

The situation is further complicated by the debate as to which TSC manifestations require two hits or loss of heterozygosity to occur. In a study of 111 TSC-associated tissues, two-thirds of hamartomas were found to contain two hits to *TSC1/TSC2*, including most SEGAs, SENs, and renal angiomyolipomas, as well as 35% of tubers [119]. The two-hit model has also been reported in two TSC patients with HME, where a germline variant in *TSC1/TSC2* was found in blood and a second somatic variant in the same gene was found in brain-derived samples [107]. Similar findings have also been reported for facial angiofibromas, where half of individuals with *TSC1/TSC2* pathogenic variants also presented with a second hit in the affected tissue as well as renal AMLs and LAM [116,120,121]. 

While the two-hit hypothesis is the prevailing model, not all TSC-associated hamartomas have been found to contain dual inactivating variants in *TSC1/TSC2*. Most of the debate surrounds whether the model applies to cortical tubers, which are thought to be epileptogenic foci in TSC. Multiple studies have proposed that while a second inactivating variant contributes to tuber pathology, it is not necessary for tuber formation. For example, Zhou and colleagues identified a single *TSC2* somatic variant in a cortical tuber in an individual with refractory epilepsy, consistent with the aforementioned finding by Martin and colleagues that only 35% of tubers were affected by second-hit variants [61,119]. While epigenetic silencing and methylation of *TSC1/TSC2* have been proposed as explanations for one-hit tumors, Martin and colleagues did not find any evidence of promoter methylation in 63 TSC-associated tissue samples [119]. They postulated that monoallelic inactivation may be sufficient for cortical tuber formation, as tubers develop prenatally and do not grow appreciably over time. However, in a study of children with surgically resected mild MCD, FCD I, FCD II, or HME, VAFs of *TSC1/TSC2* in those with FCD IIb cases were found to be strongly enriched in pools of dysmorphic neurons and balloon cells (up to 45%) compared with glial cells and morphologically normal neurons (down to 1.4%) [11]. Furthermore, the wild-type allele was nearly undetectable in a pool of 250 DNs, while both wild-type and mutant variants were clearly detected in pools of 300 morphologically normal neurons and 300 glial cells [11]. This could suggest that the inability to detect a second hit may be due to a low density of dysmorphic neurons and balloon cells in the sequenced sample.

As for other mTORopathies, dual hits in *DEPDC5* have been found in six patients with FCD [11,68,122,123,124,125]. A dual-pathway two-hit genetic model has also been proposed for FCD type IIa and drug-resistant epilepsy, where an individual presented with a germline variant in *NPRL3* and an additional somatic variant in *WNT2* in brain-derived tissue [126]. If found in other cases of MCDs, this could extend the two-hit model to genes in different but intersecting pathways and potentially explain the origin of some one-hit cases in which only *TSC1/TSC2* were sequenced. Another proposed cause for the lack of a second inactivating variant is the loss of heterozygosity due to nonhomologous recombination, an event which may be difficult to detect if tumor cell enrichment is low in the sequenced sample [117]. Overall, mosaic variants are thought to explain some of the differences in expressivity in TSC, potentially explaining a portion of milder NMI phenotypes.

## 6. Advances in Detection of Pathogenic Variants in TSC and mTORopathies

In recent years, significant strides have been made in the detection of pathogenic variants associated with TSC and mTORopathies, owing to the rapid advancements in genomic technologies and bioinformatics tools. While clinically accredited genetic laboratories report only variants with VAF greater than 10 to 20%, research laboratories have developed tools and pipelines to accurately detect mosaic variants with VAF < 1%. This is important, as TSC samples with low VAF (<5%) have been frequently reported in NMI samples [110,127]. Novel samples have also been tested with the goal of maximizing VAFs and ensuring that they represent those of the underlying pathology. Together, improved samples and detection methods have been integrated into workflows that are better able to detect mosaicism, CNVs, intronic variants, and assess the impact of these variants.

Blasco-Pérez and colleagues developed such a pipeline for the identification of pathogenic variants in TSC [128]. The first step involved multiplex ligation-dependent probe amplification (MLPA) for the identification of CNVs, and NGS using capture sequencing of *TSC1/TSC2* exons and introns, with an average coverage of >400×. Capture sequencing involves the fragmentation and capturing of target areas of interest using probes, while amplicon sequencing involves amplification of DNA fragments of interest using custom primers prior to sequencing [129,130]. In addition to variant validation with Sanger sequencing, candidate CNVs were tested for their breakpoints using long-range polymerase chain reaction (PCR), candidate splicing variants were investigated through complementary DNA (cDNA) studies, and mosaic variants with VAF < 20% were validated using amplicon-based deep sequencing (ADS) with a minimum coverage of 7000×. It should be noted that typical ES and GS use 100–150× and 30–60× read depths, respectively [68,131]. The key highlights of this workflow are the increased sequencing depths for the detection of low VAFs at both the initial and validation steps, as well as the inclusion of CNVs and intronic variants. 

Other studies of TSC and mTORopathies have developed similar pipelines with technical variations (Table 2). For example, Baldassari and colleagues also used a capture sequencing method to detect mosaicism in *TSC1* and *TSC2* [11]. However, droplet digital PCR (ddPCR), a sensitive and specific technique that allows absolute quantification of DNA, was also carried out in addition to ADS to improve estimates of low VAF [132]. Another proposed method for mosaic variant confirmation is allele-specific oligonucleotide PCR (ASO-PCR), a method which has better sensitivity than Sanger sequencing but is unable to quantify variants like ddPCR [133,134]. Klonowska and colleagues recently developed multiplex high-sensitivity PCR amplification (MHPA), an ultra-sensitive amplification method with a median read depth of 100,000× enabling detection of VAF < 0.1% [105]. 

Over the years, different tissue samples have also been used for the detection of mosaic variants in patients affected by mTORopathies. Overall, greater success has been achieved detecting mosaic variants in affected samples, such as resected brain, LAM, angiofibroma, or AML tissue [11,105,116,120]. Patients do not always receive surgical intervention, and even if they do, it is useful to identify pathogenic variants prior to surgery. Thus, the detection of variants in other samples is valuable for clinical applications. While blood, saliva, and buccal samples can be used to detect mosaic variants affecting multiple systems, isolated mosaic variants, particularly those in the brain, are unlikely to be detected using these methods. Cell-free DNA from cerebrospinal fluid (CSF) as well as DNA from trace brain tissue on stereo-electroencephalogram (SEEG) electrodes have been proposed as less invasive methods in these cases [135,136]. However, they are both still likely to be affected by low DNA yield [66]. Generally, it should be emphasized that sequencing at a high depth can result in false positives due to the higher sensitivity of the method. Methods used to preserve tissues, such as those in full-thickness frozen (FFZ) and formalin-fixed paraffin-embedded (FFPE) tissue can also lead to the generation of artifactual low VAF variants. Thus, it is important to perform technical replicates, most ideally, or orthogonal validation methods such as ddPCR or site-specific ADS to improve positive predictive value [68]. 

With the inevitable lowering of the mosaic reporting threshold in the clinic, multiple implications will result for TSC patients. Firstly, the number of *TSC1/TSC2* variants reported is likely to increase in TSC patients as well as non-TSC patients. A recent study with 100 controls not suspected to have TSC identified four mosaic variants in *TSC1/TSC2* above 3% VAF [137]. While not suspected to be pathogenic using the ACMG guidelines for germline variants, this suggests that an increased number of variants in non-TSC individuals will arise, requiring more work for their interpretation. Additionally, the ACMG/AMP guidelines do not fully address the interpretation of mosaic variants, and guidelines have only recently been released by the Clinical Genome Resource Brain Malformation Variant Curation Expert Panel for *AKT3*, *MTOR*, *PIK3CA*, and *PIK3R2* [66,138]. Mosaic variants in these genes are frequently detected in FCD II, HME, and related epilepsy conditions. The researchers found that 24 of the 28 ACMG/AMP criteria needed to be modified, taking into account VAFs and both somatic and germline evidence, as well as the delineation of phenotypic manifestations based on variable tissue expression [138].

**Table 2:** Selected studies highlighting the use of different DNA sources and assay types for the detection of mosaic variants in mTORopathies with a focus on TSC.

Study	Summary of Relevant Methodologies for the Detection for Mosaicism	Sample Type	Advantages of Method and/or Sample Used
An Integral Approach to the Molecular Diagnosis of Tuberous Sclerosis Complex: the role of mosaicism and splicing variants [128]	Three NGS panels: (1) a panel of amplicons with *TSC1* and *TSC2* exons, exon–intron boundaries, and intronic pathogenic variants, (2) an exonic capture panel, and (3) another similar in-house panel. Average coverage of >400×. Confirmation of mosaic variants by ADS at >7000× coverage.	Blood, buccal swab, saliva, or affected tissue from skin	NGS panels were able to detect variants with >1% VAF and incorporate intronic variants.
Dissecting the genetic basis of focal cortical dysplasia: a large cohort study [11]	Hybrid capture sequencing of coding exons and exon-flanking junctions in three panels with mTOR pathway and FCD candidate genes. Confirmation of pathogenic variants with ADS at ≥9000× mean read depth and ddPCR for low-VAF variants.	Affected brain tissue and blood	Two methods of orthogonal validation were used for mosaic variants, where ddPCR allowed better quantification of DNA.
Low-level mosaicism in tuberous sclerosis complex in four unrelated patients: comparison of clinical characteristics and diagnostic pathways [133]	Coding exons and flanking intronic sequences were sequenced with >30-fold coverage. ASO-PCR and ddPCR were used to confirm detected mosaic variants.	Blood, buccal mucosa, unaffected skin, and angiofibroma	ASO-PCR was found to be able to be used in place of ddPCR if unavailable, as it has similar sensitivity. However, it does not allow quantification.
Comprehensive genetic and phenotype analysis of 95 individuals with mosaic tuberous sclerosis complex [105]	Deep MPS with a ≥500× median read depth. Validation by amplicon MPS or MHPA assay with a median read depth 100,000×.	Buccal sample, saliva, blood, normal skin, normal kidney, normal lymph node, fetal tissue, urine, semen, SEGA, angiofibroma, fibrous cephalic plaque, angiomyolipoma, shagreen patch, ungual fibroma, hypomelanotic macule	Use of many sample types and high read depth of the MHPA assay allowed the detection of variants with <0.1% VAF.
Precise detection of low-level somatic variants in resected epilepsy brain tissue [68]	Deep sequencing of up to 28 epilepsy-related genes with average read depth of 1112× followed by site-specific ADS and deep-sequencing technical replicates.	Brain samples from intractable epilepsy patients	Deep sequencing technical replicates were shown to improve positive predictive values and limit false positives, especially in processed brain-derived tissue.
Detection of TSC1/TSC2 mosaic variants in patients with cardiac rhabdomyoma and tuberous sclerosis complex by hybrid-capture next-generation sequencing [139]	Hybrid capture sequencing of coding sequences and 10 kb upstream and downstream, with mean sequencing depth of 7423× in target regions. Confirmation of detected variants by ddPCR.	Umbilical cord and fetal cardiac rhabdomyoma tissue	Utilization of cord blood and fetal tissue allowed improved accuracy of prenatal TSC diagnosis.
Somatic mosaic variant gradient detected in trace brain tissue from stereo-EEG depth electrodes [136]	Trace DNA from SEEG electrodes pooled into three spatial brain groups. VAF in each group quantified with 200-fold exome sequencing and ddPCR (controlled with brain-specific glial fibrillary acidic protein) to correlate mosaic gradients with epileptogenicity.	Trace brain tissue from SEEG depth electrodes	Utilization of small amounts of DNA from electrode tissue traces allowed a molecular diagnosis and the mosaic gradient to be determined in individuals with TSC-epilepsy.
Cerebrospinal fluid liquid biopsy for detecting somatic mosaicism in brain [135]	ddPCR was employed to quantify brain-derived cell-free DNA. CSF cell-free DNA was compared to brain-specific DNA methylation patterns to determine their origin.	Cell-free DNA from CSF	Utilization of a CSF liquid biopsy is less invasive and more accessible compared with samples from neurosurgery or autopsy.

Abbreviations: VAF: variant allele frequency. MLPA: multiplex ligation-dependent probe amplification. NGS: next-generation sequencing. ADS: amplicon-based deep sequencing. ddPCR: droplet digital PCR. ASO-PCR: allele-specific oligonucleotide PCR. MPS: massive parallel sequencing. MHPA: multiplex high-sensitivity PCR amplification. SEGA: subependymal giant cell astrocytoma. CSF: cerebrospinal fluid. EEG: electroencephalogram. SEEG: stereo-electroencephalogram.

## 7. Genetic Counseling/Reproductive Counseling and Management in TSC

Genetic counseling plays a pivotal role in the comprehensive management of individuals with TSC. Employing minimally invasive diagnostic techniques ideally facilitates early genetic testing and result disclosure, thereby concluding the prolonged genetic diagnostic journey that some patients and their families undergo over months to years [66]. Furthermore, patients with a confirmed genetic diagnosis may become eligible for participation in clinical trials for emerging precision therapies [66]. For example, several mTOR inhibitors are currently in clinical trials for use in TSC [140,141], such as the RaRE-TS trial investigating the efficacy of rapamycin in drug-resistant epilepsy associated with TSC [142], the TSC-STEPS trial addressing sirolimus use to delay seizure onset in TSC infants [143], and the PRECISION I trial exploring the efficacy and safety of albumin-bound sirolimus in patients with malignant solid tumors harboring *TSC1* or *TSC2* alterations [144]. Under the six-tier approach for precision therapies proposed by Byrne and colleagues [145], mTOR inhibitors for the treatment of TSC-associated epilepsy and solid tumors fall within Tier 3, wherein the therapy directly targets gene dysfunction. As precision therapies belonging to higher tiers such as mRNA and gene replacement therapies become available, obtaining a confirmed genetic diagnosis will become even more important.

Despite the importance of genetic counseling for patients, in one study, only 66% of TSC patients received genetic counseling [146]. Genetic counseling assists in navigating the complex landscape of available treatments and facilitates the selection of interventions that align with the individual’s unique genetic profile. As previously discussed, evidence suggests mosaic patients generally experience less severe disease with fewer organs affected [65,79], which could inform management. For instance, individuals with predominantly neurological manifestations may benefit from targeted therapies aimed at managing cognitive and behavioral symptoms, with specialized neurological assessments and surveillance to obviate the need for extensive planned workup. For instance, serial electroencephalography (EEG) monitoring is becoming an increasingly used care strategy for infantile TSC-associated epilepsy, although it is not yet universal [147,148]. The EPISTOP and PREVeNT trials aimed to evaluate the impact of early antiseizure medication (i.e., vigabatrin) guided by EEG monitoring in infants with TSC. EPISTOP revealed a significant reduction in drug-resistant epilepsy and infantile spasms, emphasizing the potential of preventive strategies in altering the natural history of severe infantile epilepsy [149]. In contrast, the PREVeNT trial reported a reduced incidence of infantile spasms but did not observe a significant impact on other seizure types or drug-resistant epilepsy [150]. Neither trial demonstrated a significant impact on developmental outcomes [149,150]. Nevertheless, both trials demonstrate the utility of early diagnosis and monitoring for TSC patients, driven by advances in genetic testing.

Understanding the origin of a TSC pathogenic variant is paramount for accurate genetic counseling and reproductive management. Tissue-specific somatic variants in TSC may result in localized manifestations, such as cortical tubers and SEN, without affecting other organs. This subset of individuals may exhibit milder symptoms and face a lower risk of transmitting the disorder to their offspring [64,65,104]. In contrast, those with germline, germline mosaic, and gonosomal mosaic variants are at a higher risk of passing TSC to their children, necessitating more comprehensive genetic counseling and family planning strategies [64,65,104]. The distinction between somatic variants with minimal potential for transmission and heritable germline variants holds profound implications for reproductive counseling. In cases where the affected individual harbors a brain-only somatic variant, the risk of recurrence in subsequent pregnancies is significantly reduced.

For parents with germline variants, the genetic counseling process becomes more complex. Firstly, a germline mosaic parent may be only mildly affected or unaffected by the TSC variant [17]. For example, Tutschek and colleagues report a case in which the father of a baby born with TSC was negative for TSC variants in blood, saliva, and skin, but positive for a pathogenic *TSC2* variant in 10% of tested sperm DNA [151]. Chen and colleagues report a similar case where a fetus developed rhabdomyoma from an inherited *TSC2* variant with a paternal VAF of 7% in blood leukocytes and 20% in sperm [152]. Evidently, the risk of transmitting a pathogenic allele depends on the gonadal variant allele frequency, which can be highly variable and testable only in males through sperm analysis [109], further complicating transmission risk estimates and reproductive counseling. Genetic counselors and geneticists play a crucial role in helping parents understand the implications of germline variants, providing information about prenatal testing options, and assisting in making informed decisions regarding reproductive choices.

Prenatal testing, such as chorionic villus sampling (CVS) or amniocentesis, can be offered to parents with germline variants to determine whether the fetus has inherited the TSC-causing variant. Moreover, advancements in assisted reproductive technologies, such as preimplantation genetic testing (PGT), offer additional options for those aiming to conceive without passing on the TSC variant [116,153,154].

## 8. Future Directions and Conclusions

In conclusion, the elucidation of the genetic intricacies underlying TSC and related mTORopathies has undergone remarkable strides, augmenting our understanding of the molecular etiology and clinical heterogeneity associated with these disorders. The identification of *TSC1* and *TSC2* as primary causative genes has paved the way for a deeper comprehension of mTOR pathway dysregulation; further genotype–phenotype correlations have revealed nuanced associations, underscoring the importance of individual genetic profiles in prognostication and clinical management. Moreover, the recognition of mosaicism and somatic variants has added a layer of complexity to the genetic landscape of TSC, challenging conventional diagnostic paradigms and necessitating the integration of advanced sequencing technologies for comprehensive evaluation. The expanding repertoire of mTOR pathway genes implicated in TSC-related phenotypes underscores the need for a broader genetic testing framework to encompass the full spectrum of genetic contributors. As we contemplate the future directions of research in this field, a pivotal avenue lies in refining diagnostic strategies through the integration of cutting-edge genomic technologies to detect low-frequency mosaic variants and unravel the full genetic architecture of TSC.

However, challenges persist, including the interplay of genetic and environmental factors influencing clinical variability, necessitating large-scale collaborative efforts and multi-omics approaches to unravel the full complexity of TSC and related mTORopathies. While many studies have focused on a select number of genes in the mTOR pathways, new collaborations have embraced exome-wide and genome-wide strategies for exploration [66]. As we traverse the evolving landscape of TSC genetics, the translation of genetic discoveries into precision medical applications will continue to shape the trajectory of advancements in this field, ultimately improving the lives of individuals affected by TSC.

## References

[1] Ebrahimi-Fakhari D., Mann L.L., Poryo M., Graf N., von Kries R., Heinrich B., Ebrahimi-Fakhari D., Flotats-Bastardas M., Gortner L., Zemlin M. (2019). Correction to: Incidence of Tuberous Sclerosis and Age at First Diagnosis: New Data and Emerging Trends from a National, Prospective Surveillance Study. Orphanet J. Rare Dis..

[2] Northrup H., Koenig M.K., Pearson D.A., Au K.S. (2021). Tuberous Sclerosis Complex.

[3] Nabavi Nouri M., Zak M., Jain P., Whitney R. (2022). Epilepsy Management in Tuberous Sclerosis Complex: Existing and Evolving Therapies and Future Considerations. Pediatr. Neurol..

[4] Zöllner J.P., Franz D.N., Hertzberg C., Nabbout R., Rosenow F., Sauter M., Schubert-Bast S., Wiemer-Kruel A., Strzelczyk A. (2020). A Systematic Review on the Burden of Illness in Individuals with Tuberous Sclerosis Complex (TSC). Orphanet. J. Rare Dis..

[5] Amin S., Lux A., Calder N., Laugharne M., Osborne J., O’callaghan F. (2017). Causes of Mortality in Individuals with Tuberous Sclerosis Complex. Dev. Med. Child Neurol..

[6] Peron A., Au K.S., Northrup H. (2018). Genetics, Genomics, and Genotype-Phenotype Correlations of TSC: Insights for Clinical Practice. Am. J. Med. Genet. C Semin. Med. Genet..

[7] Karalis V., Bateup H.S. (2021). Current Approaches and Future Directions for the Treatment of MTORopathies. Dev. Neurosci..

[8] Moloney P.B., Cavalleri G.L., Delanty N. (2021). Epilepsy in the MTORopathies: Opportunities for Precision Medicine. Brain Commun..

[9] Nguyen L.H., Bordey A. (2021). Convergent and Divergent Mechanisms of Epileptogenesis in MTORopathies. Front. Neuroanat..

[10] Crino P.B. (2015). MTOR Signaling in Epilepsy: Insights from Malformations of Cortical Development. Cold Spring Harb. Perspect. Med..

[11] Baldassari S., Ribierre T., Marsan E., Adle-Biassette H., Ferrand-Sorbets S., Bulteau C., Dorison N., Fohlen M., Polivka M., Weckhuysen S. (2019). Dissecting the Genetic Basis of Focal Cortical Dysplasia: A Large Cohort Study. Acta Neuropathol..

[12] Mühlebner A., Bongaarts A., Sarnat H.B., Scholl T., Aronica E. (2019). New Insights into a Spectrum of Developmental Malformations Related to MTOR Dysregulations: Challenges and Perspectives. J. Anat..

[13] Abs E., Goorden S.M.I., Schreiber J., Overwater I.E., Hoogeveen-Westerveld M., Bruinsma C.F., Aganović E., Borgesius N.Z., Nellist M., Elgersma Y. (2013). TORC1-Dependent Epilepsy Caused by Acute Biallelic Tsc1 Deletion in Adult Mice. Ann. Neurol..

[14] Liu G.Y., Sabatini D.M. (2020). MTOR at the Nexus of Nutrition, Growth, Ageing and Disease. Nat. Rev. Mol. Cell Biol..

[15] Saxton R.A., Sabatini D.M. (2017). MTOR Signaling in Growth, Metabolism, and Disease. Cell.

[16] Wolfson R.L., Chantranupong L., Wyant G.A., Gu X., Orozco J.M., Shen K., Condon K.J., Petri S., Kedir J., Scaria S.M. (2017). KICSTOR Recruits GATOR1 to the Lysosome and Is Necessary for Nutrients to Regulate MTORC1. Nature.

[17] Nathan N., Keppler-Noreuil K.M., Biesecker L.G., Moss J., Darling T.N. (2017). Mosaic Disorders of the PI3K/PTEN/AKT/TSC/MTORC1 Signaling Pathway. Dermatol. Clin..

[18] Møller R.S., Weckhuysen S., Chipaux M., Marsan E., Taly V., Bebin E.M., Hiatt S.M., Prokop J.W., Bowling K.M., Mei D. (2016). Germline and Somatic Mutations in the MTOR Gene in Focal Cortical Dysplasia and Epilepsy. Neurol. Genet..

[19] Nakashima M., Saitsu H., Takei N., Tohyama J., Kato M., Kitaura H., Shiina M., Shirozu H., Masuda H., Watanabe K. (2015). Somatic Mutations in the MTOR Gene Cause Focal Cortical Dysplasia Type IIb. Ann. Neurol..

[20] Crino P.B. (2016). The MTOR Signalling Cascade: Paving New Roads to Cure Neurological Disease. Nat. Rev. Neurol..

[21] Orloff M.S., He X., Peterson C., Chen F., Chen J.-L., Mester J.L., Eng C. (2013). Germline PIK3CA and AKT1 Mutations in Cowden and Cowden-like Syndromes. Am. J. Hum. Genet..

[22] Carpten J.D., Faber A.L., Horn C., Donoho G.P., Briggs S.L., Robbins C.M., Hostetter G., Boguslawski S., Moses T.Y., Savage S. (2007). A Transforming Mutation in the Pleckstrin Homology Domain of AKT1 in Cancer. Nature.

[23] Manning B.D., Toker A. (2017). AKT/PKB Signaling: Navigating the Network. Cell.

[24] Poduri A., Evrony G.D., Cai X., Elhosary P.C., Beroukhim R., Lehtinen M.K., Hills L.B., Heinzen E.L., Hill A., Hill R.S. (2012). Somatic Activation of AKT3 Causes Hemispheric Developmental Brain Malformations. Neuron.

[25] Rivière J.-B., Mirzaa G.M., O’Roak B.J., Beddaoui M., Alcantara D., Conway R.L., St-Onge J., Schwartzentruber J.A., Gripp K.W., Nikkel S.M. (2012). De Novo Germline and Postzygotic Mutations in AKT3, PIK3R2 and PIK3CA Cause a Spectrum of Related Megalencephaly Syndromes. Nat. Genet..

[26] Janku F., Tsimberidou A.M., Garrido-Laguna I., Wang X., Luthra R., Hong D.S., Naing A., Falchook G.S., Moroney J.W., Piha-Paul S.A. (2011). PIK3CA Mutations in Patients with Advanced Cancers Treated with PI3K/AKT/MTOR Axis Inhibitors. Mol. Cancer Ther..

[27] Nakamura K., Kato M., Tohyama J., Shiohama T., Hayasaka K., Nishiyama K., Kodera H., Nakashima M., Tsurusaki Y., Miyake N. (2014). AKT3 and PIK3R2 Mutations in Two Patients with Megalencephaly-Related Syndromes: MCAP and MPPH. Clin. Genet..

[28] Lee W.S., Macdonald-Laurs E., Stephenson S., D’Arcy C., Maixner W., Harvey A.S., Lockhart P.J., Leventer R.J. (2023). Pathogenic RHEB Somatic Variant in a Child With Tuberous Sclerosis Complex Without Pathogenic Variants in TSC1 or TSC2. Neurology.

[29] Parmar N., Tamanoi F. (2010). Rheb G-Proteins and the Activation of MTORC1. Enzymes.

[30] Huang J., Manning B.D. (2008). The TSC1-TSC2 Complex: A Molecular Switchboard Controlling Cell Growth. Biochem. J..

[31] Alfaiz A.A., Micale L., Mandriani B., Augello B., Pellico M.T., Chrast J., Xenarios I., Zelante L., Merla G., Reymond A. (2014). TBC1D7 Mutations Are Associated with Intellectual Disability, Macrocrania, Patellar Dislocation, and Celiac Disease. Hum. Mutat..

[32] Capo-Chichi J.-M., Tcherkezian J., Hamdan F.F., Décarie J.C., Dobrzeniecka S., Patry L., Nadon M.-A., Mucha B.E., Major P., Shevell M. (2013). Disruption of TBC1D7, a Subunit of the TSC1-TSC2 Protein Complex, in Intellectual Disability and Megalencephaly. J. Med. Genet..

[33] Martin C., Meloche C., Rioux M.-F., Nguyen D.K., Carmant L., Andermann E., Gravel M., Cossette P. (2014). A Recurrent Mutation in DEPDC5 Predisposes to Focal Epilepsies in the French-Canadian Population. Clin. Genet..

[34] Scheffer I.E., Heron S.E., Regan B.M., Mandelstam S., Crompton D.E., Hodgson B.L., Licchetta L., Provini F., Bisulli F., Vadlamudi L. (2014). Mutations in Mammalian Target of Rapamycin Regulator DEPDC5 Cause Focal Epilepsy with Brain Malformations. Ann. Neurol..

[35] Nascimento F.A., Borlot F., Cossette P., Minassian B.A., Andrade D.M. (2015). Two Definite Cases of Sudden Unexpected Death in Epilepsy in a Family with a DEPDC5 Mutation. Neurol. Genet..

[36] Ververi A., Zagaglia S., Menzies L., Baptista J., Caswell R., Baulac S., Ellard S., Lynch S., Jacques T.S., Genomics England Research Consortium (2023). Germline Homozygous Missense DEPDC5 Variants Cause Severe Refractory Early-Onset Epilepsy, Macrocephaly and Bilateral Polymicrogyria. Hum. Mol. Genet..

[37] Baldassari S., Licchetta L., Tinuper P., Bisulli F., Pippucci T. (2016). GATOR1 Complex: The Common Genetic Actor in Focal Epilepsies. J. Med. Genet..

[38] Ricos M.G., Hodgson B.L., Pippucci T., Saidin A., Ong Y.S., Heron S.E., Licchetta L., Bisulli F., Bayly M.A., Hughes J. (2016). Mutations in the Mammalian Target of Rapamycin Pathway Regulators NPRL2 and NPRL3 Cause Focal Epilepsy. Ann. Neurol..

[39] Weckhuysen S., Marsan E., Lambrecq V., Marchal C., Morin-Brureau M., An-Gourfinkel I., Baulac M., Fohlen M., Kallay Zetchi C., Seeck M. (2016). Involvement of GATOR Complex Genes in Familial Focal Epilepsies and Focal Cortical Dysplasia. Epilepsia.

[40] Iffland P.H., Everett M.E., Cobb-Pitstick K.M., Bowser L.E., Barnes A.E., Babus J.K., Romanowski A.J., Baybis M., Elziny S., Puffenberger E.G. (2022). NPRL3 Loss Alters Neuronal Morphology, MTOR Localization, Cortical Lamination and Seizure Threshold. Brain.

[41] Ramaswamy S., Nakamura N., Vazquez F., Batt D.B., Perera S., Roberts T.M., Sellers W.R. (1999). Regulation of G1 Progression by the PTEN Tumor Suppressor Protein Is Linked to Inhibition of the Phosphatidylinositol 3-Kinase/Akt Pathway. Proc. Natl. Acad. Sci. USA.

[42] Tsujita Y., Mitsui-Sekinaka K., Imai K., Yeh T.-W., Mitsuiki N., Asano T., Ohnishi H., Kato Z., Sekinaka Y., Zaha K. (2016). Phosphatase and Tensin Homolog (PTEN) Mutation Can Cause Activated Phosphatidylinositol 3-Kinase δ Syndrome-like Immunodeficiency. J. Allergy Clin. Immunol..

[43] Zhou X.-P., Waite K.A., Pilarski R., Hampel H., Fernandez M.J., Bos C., Dasouki M., Feldman G.L., Greenberg L.A., Ivanovich J. (2003). Germline PTEN Promoter Mutations and Deletions in Cowden/Bannayan-Riley-Ruvalcaba Syndrome Result in Aberrant PTEN Protein and Dysregulation of the Phosphoinositol-3-Kinase/Akt Pathway. Am. J. Hum. Genet..

[44] Shao D.D., Achkar C.M., Lai A., Srivastava S., Doan R.N., Rodan L.H., Chen A.Y., Poduri A., Yang E., Brain Development Study Group (2020). Polymicrogyria Is Associated With Pathogenic Variants in PTEN. Ann. Neurol..

[45] Chen C.-Y., Chen J., He L., Stiles B.L. (2018). PTEN: Tumor Suppressor and Metabolic Regulator. Front. Endocrinol..

[46] Aerden M., Vallaeys L., Holvoet M., De Waele L., Van Den Bogaert K., Devriendt K. (2021). Homozygous Missense STRADA Mutation in a Patient with Polyhydramnios, Megalencephaly and Symptomatic Epilepsy Syndrome. Clin. Dysmorphol..

[47] Bi W., Glass I.A., Muzny D.M., Gibbs R.A., Eng C.M., Yang Y., Sun A. (2016). Whole Exome Sequencing Identifies the First STRADA Point Mutation in a Patient with Polyhydramnios, Megalencephaly, and Symptomatic Epilepsy Syndrome (PMSE). Am. J. Med. Genet. A.

[48] Basel-Vanagaite L., Hershkovitz T., Heyman E., Raspall-Chaure M., Kakar N., Smirin-Yosef P., Vila-Pueyo M., Kornreich L., Thiele H., Bode H. (2013). Biallelic SZT2 Mutations Cause Infantile Encephalopathy with Epilepsy and Dysmorphic Corpus Callosum. Am. J. Hum. Genet..

[49] Tsuchida N., Nakashima M., Miyauchi A., Yoshitomi S., Kimizu T., Ganesan V., Teik K.W., Ch’ng G.-S., Kato M., Mizuguchi T. (2018). Novel Biallelic SZT2 Mutations in 3 Cases of Early-Onset Epileptic Encephalopathy. Clin. Genet..

[50] Cattelani C., Lesiak D., Liebscher G., Singer I.I., Stasyk T., Wallnöfer M.H., Heberle A.M., Corti C., Hess M.W., Pfaller K. (2021). The SZT2 Interactome Unravels New Functions of the KICSTOR Complex. Cells.

[51] Baple E.L., Maroofian R., Chioza B.A., Izadi M., Cross H.E., Al-Turki S., Barwick K., Skrzypiec A., Pawlak R., Wagner K. (2014). Mutations in KPTN Cause Macrocephaly, Neurodevelopmental Delay, and Seizures. Am. J. Hum. Genet..

[52] Pacio Miguez M., Santos-Simarro F., García-Miñaúr S., Velázquez Fragua R., Del Pozo Á., Solís M., Jiménez Rodríguez C., Rufo-Rabadán V., Fernandez V.E., Rueda I. (2020). Pathogenic Variants in KPTN, a Rare Cause of Macrocephaly and Intellectual Disability. Am. J. Med. Genet. A.

[53] Pajusalu S., Reimand T., Õunap K. (2015). Novel Homozygous Mutation in KPTN Gene Causing a Familial Intellectual Disability-Macrocephaly Syndrome. Am. J. Med. Genet. A.

[54] van Slegtenhorst M., de Hoogt R., Hermans C., Nellist M., Janssen B., Verhoef S., Lindhout D., van den Ouweland A., Halley D., Young J. (1997). Identification of the Tuberous Sclerosis Gene *TSC1* on Chromosome 9q34. Science.

[55] Ramlaul K., Fu W., Li H., de Martin Garrido N., He L., Trivedi M., Cui W., Aylett C.H.S., Wu G. (2021). Architecture of the Tuberous Sclerosis Protein Complex. J. Mol. Biol..

[56] Santiago Lima A.J., Hoogeveen-Westerveld M., Nakashima A., Maat-Kievit A., van den Ouweland A., Halley D., Kikkawa U., Nellist M. (2014). Identification of Regions Critical for the Integrity of the TSC1-TSC2-TBC1D7 Complex. PLoS ONE.

[57] GnomAD. https://gnomad.broadinstitute.org/gene/ENSG00000165699?dataset=gnomad_r4.

[58] Rosengren T., Nanhoe S., de Almeida L.G.D., Schönewolf-Greulich B., Larsen L.J., Hey C.A.B., Dunø M., Ek J., Risom L., Nellist M. (2020). Mutational Analysis of TSC1 and TSC2 in Danish Patients with Tuberous Sclerosis Complex. Sci. Rep..

[59] Hoogeveen-Westerveld M., Ekong R., Povey S., Mayer K., Lannoy N., Elmslie F., Bebin M., Dies K., Thompson C., Sparagana S.P. (2013). Functional Assessment of TSC2 Variants Identified in Individuals with Tuberous Sclerosis Complex. Hum. Mutat..

[60] Fokkema I.F.A.C., Kroon M., López Hernández J.A., Asscheman D., Lugtenburg I., Hoogenboom J., den Dunnen J.T. (2021). The LOVD3 Platform: Efficient Genome-Wide Sharing of Genetic Variants. Eur. J. Hum. Genet..

[61] Zhou Y., Wang X., Wang J., Ding Y., Wang Y., Li H., Zhao R., Wu B. (2021). Identification of TSC2 Mosaic Mutation Limited to Cortical Tuber with TSC Targeted Sequencing: A Case Report and Literature Review. Childs. Nerv. Syst..

[62] Ding Y., Wang J., Zhou S., Zhou Y., Zhang L., Yu L., Wang Y. (2020). Genotype and Phenotype Analysis of Chinese Children With Tuberous Sclerosis Complex: A Pediatric Cohort Study. Front. Genet..

[63] Avgeris S., Fostira F., Vagena A., Ninios Y., Delimitsou A., Vodicka R., Vrtel R., Youroukos S., Stravopodis D.J., Vlassi M. (2017). Mutational Analysis of TSC1 and TSC2 Genes in Tuberous Sclerosis Complex Patients from Greece. Sci. Rep..

[64] Au K.S., Williams A.T., Roach E.S., Batchelor L., Sparagana S.P., Delgado M.R., Wheless J.W., Baumgartner J.E., Roa B.B., Wilson C.M. (2007). Genotype/Phenotype Correlation in 325 Individuals Referred for a Diagnosis of Tuberous Sclerosis Complex in the United States. Genet. Med..

[65] Tyburczy M.E., Dies K.A., Glass J., Camposano S., Chekaluk Y., Thorner A.R., Lin L., Krueger D., Franz D.N., Thiele E.A. (2015). Mosaic and Intronic Mutations in TSC1/TSC2 Explain the Majority of TSC Patients with No Mutation Identified by Conventional Testing. PLoS Genet..

[66] D’Gama A.M., Poduri A. (2023). Brain Somatic Mosaicism in Epilepsy: Bringing Results Back to the Clinic. Neurobiol. Dis..

[67] Biesecker L.G., Spinner N.B. (2013). A Genomic View of Mosaicism and Human Disease. Nat. Rev. Genet..

[68] Sim N.S., Ko A., Kim W.K., Kim S.H., Kim J.S., Shim K.-W., Aronica E., Mijnsbergen C., Spliet W.G.M., Koh H.Y. (2019). Precise Detection of Low-Level Somatic Mutation in Resected Epilepsy Brain Tissue. Acta Neuropathol..

[69] Moog U., Felbor U., Has C., Zirn B. (2020). Disorders Caused by Genetic Mosaicism. Dtsch. Arztebl. Int..

[70] Domogala D.D., Gambin T., Zemet R., Wu C.W., Schulze K.V., Yang Y., Wilson T.A., Machol I., Liu P., Stankiewicz P. (2021). Detection of Low-Level Parental Somatic Mosaicism for Clinically Relevant SNVs and Indels Identified in a Large Exome Sequencing Dataset. Hum. Genom..

[71] Walker S., Lamoureux S., Khan T., Joynt A.C.M., Bradley M., Branson H.M., Carter M.T., Hayeems R.Z., Jagiello L., Marshall C.R. (2021). Genome Sequencing for Detection of Pathogenic Deep Intronic Variation: A Clinical Case Report Illustrating Opportunities and Challenges. Am. J. Med. Genet. A.

[72] Riepe T.V., Khan M., Roosing S., Cremers F.P.M., ’t Hoen P.A.C. (2021). Benchmarking Deep Learning Splice Prediction Tools Using Functional Splice Assays. Hum. Mutat..

[73] Jaganathan K., Kyriazopoulou Panagiotopoulou S., McRae J.F., Darbandi S.F., Knowles D., Li Y.I., Kosmicki J.A., Arbelaez J., Cui W., Schwartz G.B. (2019). Predicting Splicing from Primary Sequence with Deep Learning. Cell.

[74] West H.D., Nellist M., Brouwer R.W.W., van den Hout-van Vroonhoven M.C.G.N., de Almeida L.G.D., Hendriks F., Elfferich P., Raja M., Giles P., Alfano R.M. (2023). Targeted Genomic Sequencing of *TSC1* and *TSC2* Reveals Causal Variants in Individuals for Whom Previous Genetic Testing for Tuberous Sclerosis Complex Was Normal. Hum. Mutat..

[75] Weisschuh N., Mazzola P., Zuleger T., Schaeferhoff K., Kühlewein L., Kortüm F., Witt D., Liebmann A., Falb R., Pohl L. (2023). Diagnostic Genome Sequencing Improves Diagnostic Yield: A Prospective Single-Centre Study in 1000 Patients with Inherited Eye Diseases. J. Med. Genet..

[76] Dvaladze A., Tavares E., Di Scipio M., Nimmo G., Grudzinska-Pechhacker M.K., Paton T., Tumber A., Li S., Eileen C., Ertl-Wagner B. (2022). Deep Intronic Variant in MVK as a Cause for Mevalonic Aciduria Initially Presenting as Non-Syndromic Retinitis Pigmentosa. Clin. Genet..

[77] Richards S., Aziz N., Bale S., Bick D., Das S., Gastier-Foster J., Grody W.W., Hegde M., Lyon E., Spector E. (2015). Standards and Guidelines for the Interpretation of Sequence Variants: A Joint Consensus Recommendation of the American College of Medical Genetics and Genomics and the Association for Molecular Pathology. Genet. Med..

[78] Di Scipio M., Tavares E., Deshmukh S., Audo I., Green-Sanderson K., Zubak Y., Zine-Eddine F., Pearson A., Vig A., Tang C.Y. (2020). Phenotype Driven Analysis of Whole Genome Sequencing Identifies Deep Intronic Variants That Cause Retinal Dystrophies by Aberrant Exonization. Invest. Ophthalmol. Vis. Sci..

[79] Qin W., Kozlowski P., Taillon B.E., Bouffard P., Holmes A.J., Janne P., Camposano S., Thiele E., Franz D., Kwiatkowski D.J. (2010). Ultra Deep Sequencing Detects a Low Rate of Mosaic Mutations in Tuberous Sclerosis Complex. Hum. Genet..

[80] Kwiatkowski D.J., Whittemore V.H., Thiele E.A. (2011). Tuberous Sclerosis Complex: Genes, Clinical Features and Therapeutics.

[81] Ogórek B., Hamieh L., Hulshof H.M., Lasseter K., Klonowska K., Kuijf H., Moavero R., Hertzberg C., Weschke B., Riney K. (2020). TSC2 Pathogenic Variants Are Predictive of Severe Clinical Manifestations in TSC Infants: Results of the EPISTOP Study. Genet. Med..

[82] Domínguez-Valdez L.F., Hernández-Utrera J.E., Chávez-Sánchez I.N., Peralta-Amaro A.L., Talin-Bosquez M.J., García-Pedraza L.A., Hernández-Jiménez C.A., Delgado-Carmona D.K., Gracia-Ramos A.E. (2023). Late Diagnosis of Tuberous Sclerosis: A Case Report. Oxf. Med. Case Rep..

[83] Seibert D., Hong C.-H., Takeuchi F., Olsen C., Hathaway O., Moss J., Darling T.N. (2011). Recognition of Tuberous Sclerosis in Adult Women: Delayed Presentation with Life-Threatening Consequences. Ann. Intern. Med..

[84] Sancak O., Nellist M., Goedbloed M., Elfferich P., Wouters C., Maat-Kievit A., Zonnenberg B., Verhoef S., Halley D., van den Ouweland A. (2005). Mutational Analysis of the TSC1 and TSC2 Genes in a Diagnostic Setting: Genotype--Phenotype Correlations and Comparison of Diagnostic DNA Techniques in Tuberous Sclerosis Complex. Eur. J. Hum. Genet..

[85] Benvenuto G., Li S., Brown S.J., Braverman R., Vass W.C., Cheadle J.P., Halley D.J., Sampson J.R., Wienecke R., DeClue J.E. (2000). The Tuberous Sclerosis-1 (TSC1) Gene Product Hamartin Suppresses Cell Growth and Augments the Expression of the TSC2 Product Tuberin by Inhibiting Its Ubiquitination. Oncogene.

[86] Alsowat D., Whitney R., Hewson S., Jain P., Chan V., Kabir N., Amburgey K., Noone D., Lemaire M., McCoy B. (2021). The Phenotypic Spectrum of Tuberous Sclerosis Complex: A Canadian Cohort. Child. Neurol. Open.

[87] Nabbout R., Belousova E., Benedik M.P., Carter T., Cottin V., Curatolo P., Dahlin M., Amato L.D., d’Augères G.B., de Vries P.J. (2019). Epilepsy in Tuberous Sclerosis Complex: Findings from the TOSCA Study. Epilepsia Open.

[88] Wong H.T., McCartney D.L., Lewis J.C., Sampson J.R., Howe C.J., de Vries P.J. (2015). Intellectual Ability in Tuberous Sclerosis Complex Correlates with Predicted Effects of Mutations on TSC1 and TSC2 Proteins. J. Med. Genet..

[89] Mammadova D., Vecko J., Hofmann M., Schüssler S.C., Deiters L., Canda A., Wieland A.K., Gollwitzer S., Hamer H., Trollmann R. (2023). A Single-Center Observational Study on Long-Term Neurodevelopmental Outcomes in Children with Tuberous Sclerosis Complex. Orphanet. J. Rare Dis..

[90] Farach L.S., Pearson D.A., Woodhouse J.P., Schraw J.M., Sahin M., Krueger D.A., Wu J.Y., Bebin E.M., Lupo P.J., Au K.S. (2019). Tuberous Sclerosis Complex Genotypes and Developmental Phenotype. Pediatr. Neurol..

[91] Curatolo P., Moavero R., Roberto D., Graziola F. (2015). Genotype/Phenotype Correlations in Tuberous Sclerosis Complex. Semin. Pediatr. Neurol..

[92] Numis A.L., Major P., Montenegro M.A., Muzykewicz D.A., Pulsifer M.B., Thiele E.A. (2011). Identification of Risk Factors for Autism Spectrum Disorders in Tuberous Sclerosis Complex. Neurology.

[93] Kothare S.V., Singh K., Hochman T., Chalifoux J.R., Staley B.A., Weiner H.L., Menzer K., Devinsky O. (2014). Genotype/Phenotype in Tuberous Sclerosis Complex: Associations with Clinical and Radiologic Manifestations. Epilepsia.

[94] Kothare S.V., Singh K., Chalifoux J.R., Staley B.A., Weiner H.L., Menzer K., Devinsky O. (2014). Severity of Manifestations in Tuberous Sclerosis Complex in Relation to Genotype. Epilepsia.

[95] Zeng L.-H., Rensing N.R., Zhang B., Gutmann D.H., Gambello M.J., Wong M. (2011). Tsc2 Gene Inactivation Causes a More Severe Epilepsy Phenotype than Tsc1 Inactivation in a Mouse Model of Tuberous Sclerosis Complex. Hum. Mol. Genet..

[96] Chivukula S., Modiri O., Kashanian A., Babayan D., Ibrahim G.M., Weil A.G., Tu A., Wu J.Y., Mathern G.W., Fallah A. (2021). Effect of Gene Mutation on Seizures in Surgery for Tuberous Sclerosis Complex. Can. J. Neurol. Sci..

[97] Giannikou K., Martin K.R., Abdel-Azim A.G., Pamir K.J., Hougard T.R., Bagwe S., Tang Y., MacKeigan J.P., Kwiatkowski D.J., Henske E.P. (2022). Spectrum of Germline and Somatic Mitochondrial DNA Variants in Tuberous Sclerosis Complex. Front. Genet..

[98] Muto Y., Sasaki H., Sumitomo M., Inagaki H., Kato M., Kato T., Miyai S., Kurahashi H., Shiroki R. (2022). Genotype-Phenotype Correlation of Renal Lesions in the Tuberous Sclerosis Complex. Hum. Genome Var..

[99] van Eeghen A.M., Black M.E., Pulsifer M.B., Kwiatkowski D.J., Thiele E.A. (2012). Genotype and Cognitive Phenotype of Patients with Tuberous Sclerosis Complex. Eur. J. Hum. Genet..

[100] Sudarshan S., Kumar A., Gupta A., Bhari N., Sethuraman G., Kaushal T., Pradhan A., Sapra S., Gupta N., Kaur P. (2021). Mutation Spectrum of Tuberous Sclerosis Complex Patients in Indian Population. J. Pediatr. Genet..

[101] Reyna-Fabián M.E., Hernández-Martínez N.L., Alcántara-Ortigoza M.A., Ayala-Sumuano J.T., Enríquez-Flores S., Velázquez-Aragón J.A., Varela-Echavarría A., Todd-Quiñones C.G., González-Del Angel A. (2020). First Comprehensive TSC1/TSC2 Mutational Analysis in Mexican Patients with Tuberous Sclerosis Complex Reveals Numerous Novel Pathogenic Variants. Sci. Rep..

[102] Vig A., Poulter J.A., Ottaviani D., Tavares E., Toropova K., Tracewska A.M., Mollica A., Kang J., Kehelwathugoda O., Paton T. (2020). DYNC2H1 Hypomorphic or Retina-Predominant Variants Cause Nonsyndromic Retinal Degeneration. Genet. Med..

[103] Harkness J.R., Thomas H.B., Urquhart J.E., Jamieson P., O’Keefe R.T., Kingston H.M., Deshpande C., Newman W.G., Genomics England Research Consortium (2023). Deep Intronic Variant Causes Aberrant Splicing of ATP7A in a Family with a Variable Occipital Horn Syndrome Phenotype. Eur. J. Med. Genet..

[104] Kozlowski P., Roberts P., Dabora S., Franz D., Bissler J., Northrup H., Au K.S., Lazarus R., Domanska-Pakiela D., Kotulska K. (2007). Identification of 54 Large Deletions/Duplications in TSC1 and TSC2 Using MLPA, and Genotype-Phenotype Correlations. Hum. Genet..

[105] Klonowska K., Giannikou K., Grevelink J.M., Boeszoermenyi B., Thorner A.R., Herbert Z.T., Afrin A., Treichel A.M., Hamieh L., Kotulska K. (2023). Comprehensive Genetic and Phenotype Analysis of 95 Individuals with Mosaic Tuberous Sclerosis Complex. Am. J. Hum. Genet..

[106] Ye Z., Lin S., Zhao X., Bennett M.F., Brown N.J., Wallis M., Gao X., Sun L., Wu J., Vedururu R. (2022). Mosaicism in Tuberous Sclerosis Complex: Lowering the Threshold for Clinical Reporting. Hum. Mutat..

[107] D’Gama A.M., Woodworth M.B., Hossain A.A., Bizzotto S., Hatem N.E., LaCoursiere C.M., Najm I., Ying Z., Yang E., Barkovich A.J. (2017). Somatic Mutations Activating the MTOR Pathway in Dorsal Telencephalic Progenitors Cause a Continuum of Cortical Dysplasias. Cell Rep..

[108] Bizzotto S. (2023). The Human Brain through the Lens of Somatic Mosaicism. Front. Neurosci..

[109] Giannikou K., Lasseter K.D., Grevelink J.M., Tyburczy M.E., Dies K.A., Zhu Z., Hamieh L., Wollison B.M., Thorner A.R., Ruoss S.J. (2019). Low-Level Mosaicism in Tuberous Sclerosis Complex: Prevalence, Clinical Features, and Risk of Disease Transmission. Genet. Med..

[110] Treichel A.M., Hamieh L., Nathan N.R., Tyburczy M.E., Wang J.-A., Oyerinde O., Raiciulescu S., Julien-Williams P., Jones A.M., Gopalakrishnan V. (2019). Phenotypic Distinctions between Mosaic Forms of Tuberous Sclerosis Complex. Genet. Med..

[111] Vadlamudi L., Dibbens L.M., Lawrence K.M., Iona X., McMahon J.M., Murrell W., Mackay-Sim A., Scheffer I.E., Berkovic S.F. (2010). Timing of de Novo Mutagenesis—A Twin Study of Sodium-Channel Mutations. N. Engl. J. Med..

[112] Byers H.M., Jensen D.M., Glass I.A., Bennett J.T. (2018). Minimal Mosaicism, Maximal Phenotype: Discordance between Clinical and Molecular Findings in Two Patients with Tuberous Sclerosis. Am. J. Med. Genet. C Semin. Med. Genet..

[113] Lim J.S., Gopalappa R., Kim S.H., Ramakrishna S., Lee M., Kim W.-I., Kim J., Park S.M., Lee J., Oh J.-H. (2017). Somatic Mutations in TSC1 and TSC2 Cause Focal Cortical Dysplasia. Am. J. Hum. Genet..

[114] Hoelz H., Coppenrath E., Hoertnagel K., Roser T., Tacke M., Gerstl L., Borggraefe I. (2018). Childhood-Onset Epileptic Encephalopathy Associated With Isolated Focal Cortical Dysplasia and a Novel TSC1 Germline Mutation. Clin. EEG Neurosci..

[115] Fujita A., Ando K., Kobayashi E., Mitani K., Okudera K., Nakashima M., Miyatake S., Tsurusaki Y., Saitsu H., Seyama K. (2016). Detection of Low-Prevalence Somatic TSC2 Mutations in Sporadic Pulmonary Lymphangioleiomyomatosis Tissues by Deep Sequencing. Hum. Genet..

[116] Ikeda K.M., House A.A., Connaughton D.M., Pautler S.E., Siu V.M., Jones M.-L. (2021). Potential Pitfalls in Pre-Implantation Genetic Diagnosis in a Patient with Tuberous Sclerosis and Isolated Mosaicism for a TSC2 Variant in Renal Tissue. Mol. Syndromol..

[117] Treichel A.M., Boeszoermenyi B., Lee C.-C.R., Moss J., Kwiatkowski D.J., Darling T.N. (2023). Diagnosis of Mosaic Tuberous Sclerosis Complex Using Next-Generation Sequencing of Subtle or Unusual Cutaneous Findings. JID Innov..

[118] Chernoff J. (2021). The Two-Hit Theory Hits 50. Mol. Biol. Cell.

[119] Martin K.R., Zhou W., Bowman M.J., Shih J., Au K.S., Dittenhafer-Reed K.E., Sisson K.A., Koeman J., Weisenberger D.J., Cottingham S.L. (2017). The Genomic Landscape of Tuberous Sclerosis Complex. Nat. Commun..

[120] Han B., Lee J., Kwak Y.J., Kim H.-Y., Lee K.H., Shim Y., Lee H., Park S.-H. (2021). A Second Hit Somatic (p.R905W) and a Novel Germline Intron-Mutation of TSC2 Gene Is Found in Intestinal Lymphangioleiomyomatosis: A Case Report with Literature Review. Diagn. Pathol..

[121] Tyburczy M.E., Wang J.-A., Li S., Thangapazham R., Chekaluk Y., Moss J., Kwiatkowski D.J., Darling T.N. (2014). Sun Exposure Causes Somatic Second-Hit Mutations and Angiofibroma Development in Tuberous Sclerosis Complex. Hum. Mol. Genet..

[122] Baulac S., Ishida S., Marsan E., Miquel C., Biraben A., Nguyen D.K., Nordli D., Cossette P., Nguyen S., Lambrecq V. (2015). Familial Focal Epilepsy with Focal Cortical Dysplasia Due to DEPDC5 Mutations. Ann. Neurol..

[123] Mirzaa G.M., Campbell C.D., Solovieff N., Goold C., Jansen L.A., Menon S., Timms A.E., Conti V., Biag J.D., Adams C. (2016). Association of MTOR Mutations With Developmental Brain Disorders, Including Megalencephaly, Focal Cortical Dysplasia, and Pigmentary Mosaicism. JAMA Neurol..

[124] Ribierre T., Deleuze C., Bacq A., Baldassari S., Marsan E., Chipaux M., Muraca G., Roussel D., Navarro V., Leguern E. (2018). Second-Hit Mosaic Mutation in MTORC1 Repressor DEPDC5 Causes Focal Cortical Dysplasia-Associated Epilepsy. J. Clin. Investig..

[125] Lee W.S., Stephenson S.E.M., Howell K.B., Pope K., Gillies G., Wray A., Maixner W., Mandelstam S.A., Berkovic S.F., Scheffer I.E. (2019). Second-Hit DEPDC5 Mutation Is Limited to Dysmorphic Neurons in Cortical Dysplasia Type IIA. Ann. Clin. Transl. Neurol..

[126] Bennett M.F., Hildebrand M.S., Kayumi S., Corbett M.A., Gupta S., Ye Z., Krivanek M., Burgess R., Henry O.J., Damiano J.A. (2022). Evidence for a Dual-Pathway, 2-Hit Genetic Model for Focal Cortical Dysplasia and Epilepsy. Neurol. Genet..

[127] Treichel A.M., Kwiatkowski D.J., Moss J., Darling T.N. (2020). A Diagnostic Algorithm for Enhanced Detection of Mosaic Tuberous Sclerosis Complex in Adults. Br. J. Dermatol..

[128] Blasco-Pérez L., Iranzo-Nuez L., López-Ortega R., Martínez-Cruz D., Camprodon-Gómez M., Tenés A., Antolín M., Tizzano E.F., García-Arumí E. (2023). An Integral Approach to the Molecular Diagnosis of Tuberous Sclerosis Complex: The Role of Mosaicism and Splicing Variants. J. Mol. Diagn..

[129] Pel J., Leung A., Choi W.W.Y., Despotovic M., Ung W.L., Shibahara G., Gelinas L., Marziali A. (2018). Rapid and Highly-Specific Generation of Targeted DNA Sequencing Libraries Enabled by Linking Capture Probes with Universal Primers. PLoS ONE.

[130] Singh R.R. (2022). Target Enrichment Approaches for Next-Generation Sequencing Applications in Oncology. Diagnostics.

[131] Koboldt D.C. (2020). Best Practices for Variant Calling in Clinical Sequencing. Genome Med..

[132] Lee W.S., Lockhart P.J. (2023). Utility of Droplet Digital Polymerase Chain Reaction for Studying Somatic Mosaicism: Brain Malformations and Beyond. Neural Regen. Res..

[133] Manzanilla-Romero H.H., Weis D., Schnaiter S., Rudnik-Schöneborn S. (2021). Low-Level Mosaicism in Tuberous Sclerosis Complex in Four Unrelated Patients: Comparison of Clinical Characteristics and Diagnostic Pathways. Am. J. Med. Genet. A.

[134] Nellist M., Brouwer R.W.W., Kockx C.E.M., van Veghel-Plandsoen M., Withagen-Hermans C., Prins-Bakker L., Hoogeveen-Westerveld M., Mrsic A., van den Berg M.M.P., Koopmans A.E. (2015). Targeted Next Generation Sequencing Reveals Previously Unidentified TSC1 and TSC2 Mutations. BMC Med. Genet..

[135] Ye Z., Chatterton Z., Pflueger J., Damiano J.A., McQuillan L., Harvey A.S., Malone S., Do H., Maixner W., Schneider A. (2021). Cerebrospinal Fluid Liquid Biopsy for Detecting Somatic Mosaicism in Brain. Brain Commun..

[136] Ye Z., Bennett M.F., Neal A., Laing J.A., Hunn M.K., Wittayacharoenpong T., Todaro M., Patel S.K., Bahlo M., Kwan P. (2022). Somatic Mosaic Pathogenic Variant Gradient Detected in Trace Brain Tissue From Stereo-EEG Depth Electrodes. Neurology.

[137] Ye Z., Lin S., Zhao X., Wallis M., Gao X., Sun L., Wu J., Duan J., Yao Y., Li L. (2024). Are Germline Mosaic TSC1/2 Variants Present in Controls? Implications for Diagnosis. Pediatr. Neurol..

[138] Lai A., Soucy A., El Achkar C.M., Barkovich A.J., Cao Y., DiStefano M., Evenson M., Guerrini R., Knight D., Lee Y.-S. (2022). The ClinGen Brain Malformation Variant Curation Expert Panel: Rules for Somatic Variants in AKT3, MTOR, PIK3CA, and PIK3R2. Genet. Med..

[139] Wang S., Sun H., Wang J., Gu X., Han L., Wu Y., Yan H., Han L., Zhang H., He Y. (2021). Detection of TSC1/TSC2 Mosaic Variants in Patients with Cardiac Rhabdomyoma and Tuberous Sclerosis Complex by Hybrid-Capture next-Generation Sequencing. Mol. Genet. Genom. Med..

[140] Luo C., Ye W.-R., Shi W., Yin P., Chen C., He Y.-B., Chen M.-F., Zu X.-B., Cai Y. (2022). Perfect Match: MTOR Inhibitors and Tuberous Sclerosis Complex. Orphanet. J. Rare Dis..

[141] D’Gama A.M., Poduri A. (2021). Precision Therapy for Epilepsy Related to Brain Malformations. Neurotherapeutics.

[142] Clinicaltrials.gov. https://clinicaltrials.gov/study/NCT05534672.

[143] Clinicaltrials.gov. https://clinicaltrials.gov/study/NCT05104983.

[144] Iyer G., Demeure M.J., Deming D.A., Federman N., McKean M., Lee E.K., Spira A.I., Kwiatkowski D.J., Hussein M.A., Gordon E.M. (2023). Phase 2, Multicenter, Open-Label Basket Trial of Nab-Sirolimus for Patients with Malignant Solid Tumors Harboring Pathogenic Inactivating Alterations in TSC1 or TSC2 Genes (PRECISION I). J. Clin. Orthod..

[145] Byrne S., Enright N., Delanty N. (2021). Precision Therapy in the Genetic Epilepsies of Childhood. Dev. Med. Child. Neurol..

[146] Alsowat D., Zak M., McCoy B., Kabir N., Al-Mehmadi S., Chan V., Whitney R. (2020). A Review of Investigations for Patients with Tuberous Sclerosis Complex Who Were Referred to the Tuberous Sclerosis Clinic at the Hospital for Sick Children: Identifying Gaps in Surveillance. Pediatr. Neurol..

[147] Słowińska M., Kotulska K., Szymańska S., Roberds S.L., Fladrowski C., Jóźwiak S. (2021). Approach to Preventive Epilepsy Treatment in Tuberous Sclerosis Complex and Current Clinical Practice in 23 Countries. Pediatr. Neurol..

[148] Whitney R., Zak M., Haile D., Nabavi Nouri M. (2022). The State of Pediatric Tuberous Sclerosis Complex Epilepsy Care: Results from a National Survey. Epilepsia Open.

[149] Kotulska K., Kwiatkowski D.J., Curatolo P., Weschke B., Riney K., Jansen F., Feucht M., Krsek P., Nabbout R., Jansen A.C. (2021). Prevention of Epilepsy in Infants with Tuberous Sclerosis Complex in the EPISTOP Trial. Ann. Neurol..

[150] Bebin E.M., Peters J.M., Porter B.E., McPherson T.O., O’Kelley S., Sahin M., Taub K.S., Rajaraman R., Randle S.C., McClintock W.M. (2023). Early Treatment with Vigabatrin Does Not Decrease Focal Seizures or Improve Cognition in Tuberous Sclerosis Complex: The PREVeNT Trial. Ann. Neurol..

[151] Tutschek B., Mayer K., Rauch A. (2020). Fetal Tuberous Sclerosis and Diagnosis of Paternal Gonadal Mosaicism. Ultrasound Obstet. Gynecol..

[152] Chen L., Jiang Y., Wang J. (2020). Fetal Cardiac Rhabdomyoma Due to Paternal Mosaicism of TSC2: A Case Report. Medicine.

[153] Brezina P.R., Kutteh W.H. (2015). Clinical Applications of Preimplantation Genetic Testing. BMJ.

[154] Naja R.P., Dhanjal S., Doshi A., Serhal P., Delhanty J., SenGupta S.B. (2016). The Impact of Mosaicism in Preimplantation Genetic Diagnosis (PGD): Approaches to PGD for Dominant Disorders in Couples without Family History. Prenat. Diagn..

